# Cataracte morganienne

**DOI:** 10.11604/pamj.2017.28.124.13213

**Published:** 2017-10-10

**Authors:** Alsubari Alwan, Riani Mohammed

**Affiliations:** 1 Université Mohammed V, Hôpital Militaire d’Instruction Mohammed V, Rabat, Maroc

**Keywords:** Cataracte, gène visuelle, complications, Cataract, visual gene, complications

## Abstract

Cataract is a total or partial clouding of the lens of the eye. It can be congenital or acquired. Cataract associated with the aging process (senile) is the most common type. Common symptoms include visual impairment which varies based on the type and the density of lens opacity. Cataracts etiology is varied and treatment is based on surgery. In patients with morgagnian cataract cortex liquefies and lens core hardens and becomes cloudy. We here report the case of a 51-year old patient with no notable medical history presenting with progressive decreased visual acuity in the left eye with no pain or redness of the eye associated. Ophthalmologic examination showed visual acuity (the individual can see hand movement), clear cornea, normal anterior chamber depth, an intraocular pressure of 15 mmgh and morgagnian cataract. The patient underwent phacoemulsification cataract surgery with implantation within the capsular bag. Post-operative suites were simple.

## Image en médecine

La cataracte est une opacification partielle ou totale du cristallin. Elle peut être congénitale ou acquise. Celle liée au vieillissement (sénile) est la plus fréquente. Sa symptomatologie commune est le gène visuel qui est variable en fonction de la topographie et de la densité des opacités. Ses étiologies sont variées et son traitement est chirurgical. Le cortex de cataracte morganienne se liquéfie et le noyau se durci et devient foncé. Nous rapportant le cas d'un patient âgé de 51 ans sans antécédents pathologiques notable qui consulte par une baisse de l'acuité visuelle de façon progressive au niveau de l'œil gauche sans notion de douleur ou de rougeur oculaire. L'examen ophtalmologique trouve une acuité visuelle (voit la main bougé), une cornée claire, une chambre antérieur de profondeur normal, une pression intraoculaire à 15 mmgh et une cataracte morganienne. Le patient a été opéré par phacoémulsification avec mis en place d'un implant dans le sac capsulaire. Les suites post op ératoires étaient simples.

**Figure 1:**
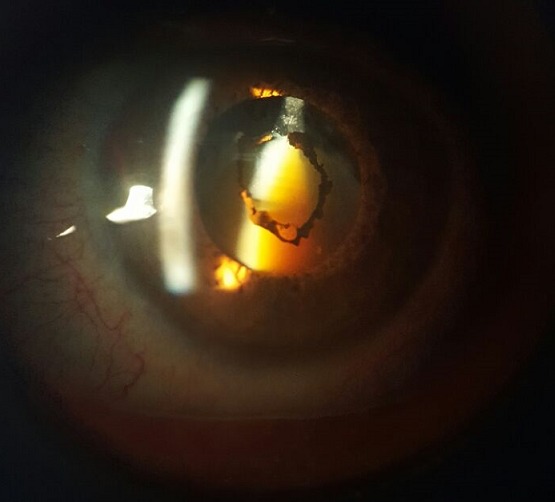
Cataracte morganienne

